# Modified Qing’e Formula protects against UV-induced skin oxidative damage *via* the activation of Nrf2/ARE defensive pathway

**DOI:** 10.3389/fphar.2022.976473

**Published:** 2022-10-28

**Authors:** Shan Zhu, Wenxiao Qin, Tao Liu, Tao Liu, Hongfei Ma, Cunyu Hu, Xiaofeng Yue, Yiqi Yan, Yingshuang Lv, Zijing Wang, Zhiyue Zhao, Xiang Wang, Yan Liu, Qingmei Xia, Han Zhang, Nan Li

**Affiliations:** ^1^ State Key Laboratory of Component Traditional Chinese Medicine, Tianjin University of Traditional Chinese Medicine, Tianjin, China; ^2^ State Key Laboratory of Formulation, Ministry of Education, Tianjin University of Traditional Chinese Medicine, Tianjin, China; ^3^ Engineering Research Center of Modern Chinese Medicine Discovery and Preparation Technique, Ministry of Education, Tianjin University of Traditional Chinese Medicine, Tianjin, China; ^4^ Tianjin University of Technology, Tianjin, China

**Keywords:** Traditional Chinese medicine (TCM), Modified Qing’e Formula (MQEF), photoaging, oxidative skin damage, Nrf2/ARE signaling pathway

## Abstract

Exposure to ultraviolet (UV) light triggers the rapid generation and accumulation of reactive oxygen species (ROS) in skin cells, which increases oxidative stress damage and leads to photoaging. Nuclear factor E2-related factor 2 (Nrf2) modulates the antioxidant defense of skin cells against environmental factors, especially ultraviolet radiation. Natural products that target Nrf2-regulated antioxidant reactions are promising candidates for anti-photoaging. The aim of this study was to investigate the protective effect of Modified Qing’e Formula (MQEF) on UV-induced skin oxidative damage and its molecular mechanisms. In this study, the photoaging models of human keratinocytes (HaCaT) and ICR mice were established by UV irradiation. *In vitro* models showed that MQEF displayed potent antioxidant activity, significantly increased cell viability and reduced apoptosis and excess ROS levels. Meanwhile, the knockdown of Nrf2 reversed the antioxidant and anti-apoptotic effects of MQEF. *In vivo* experiments indicated that MQEF could protect the skin against UV-exposed injury which manifested by water loss, sensitivity, tanning, wrinkling, and breakage of collagen and elastic fibers. The application of MQEF effectively increased the activity of antioxidant enzymes and reduced the content of malondialdehyde (MDA) in mice. In addition, MQEF was able to activate Nrf2 nuclear translocation in mouse skin tissue. In summary, MQEF may attenuate UV-induced photoaging by upregulating Nrf2 expression and enhancing antioxidant damage capacity. MQEF may be a potential candidate to prevent UV-induced photoaging by restoring redox homeostasis.

## Graphical Abstract

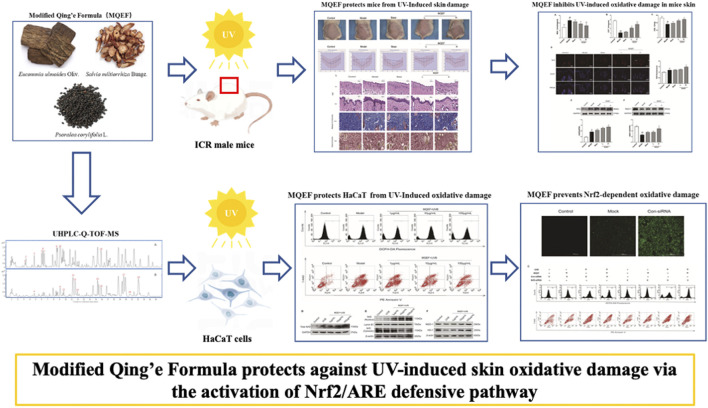

## Introduction

The skin serves as a protective barrier against the external environment, which determines its critical function in maintaining homeostasis (Harris-Tryon and Grice, 2022). Skin aging is the most visual and external manifestation of the aging of the body, and it is driven by internal and external factors that cumulatively lead to progressive changes in physiology and appearance. Intrinsic aging tends to intensify with advancing age, whereas extrinsic aging is primarily caused by exposure to ultraviolet (UV) irradiation from the Sun, also known as photoaging (Krutmann et al., 2021). The main clinical manifestations of photoaging are dry and rough skin, deepened wrinkles, sagging skin, vasodilation, and hyperpigmentation in exposed areas (Chen et al., 2021).

Accumulating evidence has shown that skin photoaging induced by UV-irradiation is associated with excessive production of reactive oxygen species (ROS), which can cause an imbalance of cellular oxygen levels, triggering oxidative stress and impairing the antioxidant defense system (Hseu et al., 2019). Nuclear factor E2-related factor 2 (Nrf2) is a redox-sensitive transcription factor that regulates cellular antioxidant defenses against environmental factors, especially ultraviolet radiation (Li et al., 2012; Torrente and Denicola., 2022). Nrf2 is a redox-sensitive basic leucine zipper protein (bZIP) and a major transcriptional enhancer known to be involved in the gene expression of distinct phase II detoxifying enzymes and cytoprotective protein-coding genes such as heme oxygenase-1 (HO-1) and NAD(P)H quinone oxidoreductase 1 (NQO-1) (Melo et al., 2021). Antioxidant-response element (ARE) is a cis-regulatory deoxyribonucleic acid (DNA) sequence located in the promoter region of phase II detoxification enzymes (Liu et al., 2022). During redox, Nrf2 will translocate to the nucleus to bind to ARE thereby regulating the transcriptional activation of antioxidant genes so as to regulate and neutralize oxidative stress (Wu et al., 2017; Wakamori et al., 2022). Nrf2 plays a central role in protecting cells from oxidative damage and maintaining cellular homeostasis. Therefore, enhancing the antioxidant capacity of skin with natural medicines targeting Nrf2 regulation may be a promising strategy for the treatment of UV-induced oxidative stress damage.

Modified Qing’e Formula (MQEF) is a traditional Chinese medicine (TCM) formulation comprising three herbs: *Eucommia ulmoides* Oliv., *Psoralea corylifolia* L., *Salvia miltiorrhiza* Bunge. It is on the basis of the ancient prescription Qing’e Formula (QEF), minus *Juglans regia* L. and *Allium sativum* L., plus *Salvia miltiorrhiza* Bunge. The QEF has been used for more than 800 years and is originally recorded in the Prescriptions of the Bureau of Taiping People’s Welfare Pharmacy during the Song Dynasty (10th century CE) with the effect of invigorating the kidney and strengthening bones, invigorating the circulation of blood and beautifying (Xiong et al., 2022). It could be used to alleviate osteoporosis in the postmenopausal woman and improve menopausal symptoms (Yang et al., 2015). Interestingly, it was found that *Salvia miltiorrhiza* Bunge. Enhanced the estrogen effect of QEF on ovariectomized rats and MQEF was more effective in the overall treatment of menopausal diseases (Zhang et al., 2016). Recent studies had shown that QEF alleviated oxidative stress damage of human skin fibroblasts induced by H_2_O_2_ (Zhong et al., 2016) and slowed down the aging process in d-galactose-induced mice (Tian et al., 2020). Moreover, our previous studies had revealed that corylin from *Psoralea corylifolia* L. and Oroxylin A from *Eucommia ulmoides* Oliv. inhibit oxidative stress by activating Nrf2 to prevent UV-induced damage (Zhao et al., 2021; Li et al., 2022; Zhu et al., 2022). However, whether MQEF could prevent UV radiation-induced photoaging remains unknown. In this study, we investigated the protective effect of MQEF on UV-induced photoaging of mice skin and Human keratinocyte (HaCaT) cells, and explored whether it is related to the mechanisms of suppressing oxidative stress by upregulating Nrf2, providing theoretical support for the clinical application of MQEF as an anti-aging remedy.

## Materials and methods

### Chemicals and plants materials

*Eucommia ulmoides* Oliv., *Psoralea corylifolia* L. and *Salvia miltiorrhiza* Bunge. Were purchased from Tongrentang (Beijing, China). The identification of Chinese medicine by the Tianjin University of Chinese medicine is valid. Ethanol, methanol, formic acid and acetonitrile were chromatographic purity and obtained from Thermo Fisher Scientific (Carlsbad, United States). Ultra-pure water was filtered by a Mingche D24 UV system (Merck Millipore). Dimethyl sulfoxide (DMSO) was attained from Sigma (Saint Louis, United States). Reagents for cell culture such as Dulbecco’s Modified Eagle Medium (DMEM), Minimum essential medium (MEM), Fetal bovine serum (FBS), phosphate buffered saline (PBS) and penicillin-streptomycin (PS) were purchased from Gibco BRL (Gaithersburg, Maryland United States). Cell-counting-kit-8 (CCK-8) kits and lactate dehydrogenase (LDH) kits were obtained from Dojindo Laboratories (Kumamoto, Japan). 2′,7' -dichlorodihydrofluorescein diacetate (DCFH-DA) reagents were attained from Sigma (Saint Louis, MO, United States). Annexin V-FITC/7-AAD apoptosis detection kits were purchased from BD Biosciences (New Jersey, FL, United States). Malondialdehyde (MDA), catalase (CAT), superoxide dismutase (SOD) and glutathione peroxidase (GSH-Px) assay kits were attained from Nanjing Jiancheng Bioengineering Institute (Nanjing, China). TRIzol reagen and cDNA Reverse Transcription Kit were attained from Ambion (Carlsbad, United States). FastStart universal SYBR Green Master (Roche, Switzerland) was used for polymerase chain reaction (PCR) reaction. PCR primers were obtained from Sangon Biotech (Shanghai, China). Radio immunoprecipitation assay (RIPA) lysate and phenylmethanesulfonyl fluoride (PMSF) protease inhibitor (Solarbio, Beijing, China) were used to extract the protein, and the protein content was quantified by bicinchoninic acid (BCA) kit (Thermo Fisher Scientific, Carlsbad, United States). The primary antibodies such as Nrf2, HO-1, NQO-1, glyceraldehyde-3-phosphate dehydrogenase (GAPDH), Lamin B1 and *β*-actin were purchased from Cell Signaling Technology (Boston, MA, United States). Small interference ribonucleic acid (siRNA) and siRNA Mate transfection reagent were attained from Jima Genetics (Suzhou, China).

### Preparation of MQEF extracts

*Eucommia ulmoides* Oliv., *Psoralea corylifolia* L., *Salvia miltiorrhiza* Bunge. Were accurately weighed in proportion according to the prescription amount of 2:1:2, heated and refluxed with 10 times of 75% ethanol for extraction twice, filtered while hot, and the extract was combined. The extracts were evaporated by rotation to flow extracted and freeze-dried in a vacuum for 24 h until it was dried. The dry pastes were ground and crushed to obtain MQEF extracts. MQEF extracts were dissolved in DMSO for cell administration. In order to facilitate the administration of drugs to animals, the prepared extracts were prepared into gel preparation and applied to the skin surface of mice.

### Instrumentation and UHPLC-Q-TOF-MS conditions

LC-MS/MS analysis was performed on an Agilent ultra-high performance liquid chromatography 1290 UPLC system with a Waters UPLC ethylene bridged hybrid (BEH) C18 column (2.1 mm × 100 mm I.D., 1.7 mm, Waters, Milford, MA, United States) (Shi et al., 2022). The column temperature was set at 35 °C and the sample injection volume was set at 3 μl. The flow rate was set at 0.3 ml/min. The mobile phase consisted of 0.1% formic acid in water (A) and 0.1% formic acid in acetonitrile (B). The multi-step linear elution gradient program was as follows: 0–20 min, 10–65% B; 20–26 min, 65–80% B. The Q-TOF-MS scan range was set at m/z 50–1,500 in both positive and negative ion modes. Drying gas (N2) flow rate was 11.0 L/min, drying gas temperature set at 350°C, nebulizer 40 psig, the capillary voltage was 3500 V, fragment was 135 V, collision energy was 30 V. Data analysis was performed using Agilent Masshunter (B.07.00).

We accurately weighted 50 mg of MQEF extracts and added 1 ml methanol to dissolve, then vortexed for 30 s. After centrifugation at 12,000 rpm for 15 min, pass through a 0.22 μm filter membrane and the sample solution was injected into the supernatant (5 µl) for further UHPLC-Q-TOF-MS analysis. Take an appropriate amount of the reference substance, precisely weigh it, and add methanol to make a reference substance solution with a mass concentration of 1 mg/ml. Take 100 μl of each reference solution to prepare a mixed reference solution.

### Cell culture, MQEF pretreatment and UVB radiation

HaCaT cells and HEK293T cells were obtained from the American Type Culture Collection (ATCC; Manassas, VA, United States) and cultured in MEM or DMEM medium containing 10% FBS, 100 mg/ml penicillin/streptomycin. Cells were cultured in a humidified atmosphere of 95% air and 5% CO_2_ at 37°C. We diluted MQEF dissolved in DMSO with fresh medium to different concentrations and treated with HaCaT cells for 24 h. Then the drug solution was then discarded and PBS was added to irradiate with UVB radiometer (SH2B, Sigma) at a dose of 60 mJ/cm^2^. Normal control group (Control), UVB irradiation group (UVB), and UVB plus MQEF group (MQEF) with different concentrations were set. The control group was neither treated with drugs nor exposed to UVB. After irradiation, PBS was replaced with fresh medium and cells were incubated in an incubator for 24 h, and then treated for subsequent analysis.

### Cell viability assay

The effects of different concentrations of MQEF on cell viability were investigated using CCK-8 kits. After cell treatment, the supernatant of the cell plate was absorbed and incubated with CCK-8 working solution at 37°C. The optical density (OD) value was measured at 450 nm of the microplate. In a physiological state, LDH existed in cells. When the cell membrane was damaged, LDH was released from the cell to the cell outside (Fotakis and Timbrell, 2006). The LDH kits were used to detect the degree of cell membrane damage. To be specific, the LDH detection buffer was added to the cell supernatant, and the stop solution was added after 15 min of incubation away from light. The OD value was measured at 490 nm with a microplate reader.

### Measurement of ROS and cell apoptosis by flow cytometry

DCFH-DA could pass through the cell membrane freely and be hydrolyzed by intracellular esterase to form DCFH that cannot pass through the cell membrane, thus accumulating in the cell. Intracellular ROS oxidized non-fluorescent DCFH to fluorescent DCF. Therefore, the ROS level could be reflected by measuring the fluorescence intensity of DCF (Krutmann et al., 2021). Using Annexin V labeled with FITC as a fluorescent probe, the occurrence of apoptosis could be detected by flow cytometry. Annexin V was matched with 7-ADD to distinguish the early apoptotic cells from late apoptotic cells and dead cells (Zembruski et al., 2012). Specifically, cultured HaCaT cells were collected on Petri dishes and divided into two sections. Part of the cells was treated with DCFH-DA for 30 min, and the fluorescence intensity was detected by flow cytometry. The other cells were suspended in a 1×Binding buffer. Annexin V and 7-AAD were added into cell suspension, and a certain amount of 1×Binding buffer was added after dark culture at room temperature for 15 min. The apoptosis level of cells was detected by flow cytometry.

### Biochemical indicator

SOD, CAT and GSH-Px kits were used to detect the enzymatic antioxidant activity, and MDA kits were used to detect the degree of lipid peroxidation. The operation method was carried out according to the kit instruction.

### Reverse transcription-polymerase chain reaction

HaCaT cells were dissolved with TRIzol reagent and total RNA was extracted according to the reagent instructions. RNA was reversely transcribed into cDNA using the kit and the reaction conditions were 25°C for 10 min, 37°C for 120 min, 85°C for 5 min and held at 4°C. Then, the cDNA was amplified for individual PCR reactions using FastStart universal SYBR Green Master, the reaction conditions were as follows: Initial denaturation at 95°C for 10 min; DNA amplification at 95°C for 10 s, 60°C for 30 s (40 cycles); and a final extension step of 65–95°C (5 s/cycle; 0.5°C/cycle) for 38 cycles. The level of mRNA was normalized to the level of GAPDH, and compared with the control group (treated with the same volume of complete DMEM) using the 2^−ΔΔCq^ method (Ma et al., 2021). The primer sequences used were as follows: Nrf2 sense, 5′-TCC​AAG​TCC​AGA​AGC​CAA​ACT​GAG-3′ and antisense, 5′-GGA​GAG​GAT​GCT​GCT​GAA​GGA​ATG-3′; NQO-1 sense, 5′-AAG​CCG​CAG​ACC​TTG​TGA​TAT​TGC-3′ and antisense, 5′-CAT​GGC​AGC​GTA​AGT​GTA​AGC​AAG​C-3′; HO-1 sense, 5′-CCT​CCC​TGT​ACC​ACA​TCT​ATC​T-3′ and antisense, 5′-GCT​CTT​CTG​GGA​AGT​AGA​CCG-3′.

### Western blot analysis

RIPA lysis buffer was used to split the sample, and the protein concentration in the sample was measured and quantified according to the BCA kit instructions. The sample proteins were added in 8–12% SDS-PAGE gel for electrophoresis and then transferred to the PVDF membrane, The membrane was sealed with 5% skimmed milk and incubated with primary antibody at 4°C and then incubated with corresponding secondary antibody at room temperature for 1 h. The intensity of the bands was visualized using enhanced chemiluminescence (ECL) reagent and analyzed using ImageJ software.

### siRNA transfection

When the cells grew to 70%, Nrf2-siRNA and siRNA mate were mixed in serum-free MEM medium and incubated at room temperature for 15 min MEM complete medium was supplemented in the transfection medium and added to the cells to inhibit Nrf2. After transfection, the transfection solution was discarded and HaCaT cells were treated with MQEF and UVB for 24 h. The levels of ROS and apoptosis were detected by the method described earlier in this paper. The Nrf2-siRNA sequences used were as follows: Nrf2-siRNA-1 sense, 5′-CAG​AAG​UUG​ACA​AUU​AUC​ATT-3′ and antisense, 5′-UGA​UAA​UUG​UCA​ACU​UCU​GTC-3′; Nrf2-siRNA-2 sense, 5′-CUG​UUG​AUU​UAG​ACG​GUA​UTT-3′ and antisense, 5′-AUA​CCG​UCU​AAA​UCA​ACA​GTT-3′; Nrf2-siRNA-3 sense, 5′-CAG​CUA​UGG​AGA​CAC​ACU​AT-3′ and antisense, 5′-UAG​UGU​GUC​UCC​AUA​GCU​GC-3′.

### Animal modeling and grouping

ICR male mice with the weight of 18–22 g were acquired from Weitong Lihua Laboratory Animal Co., Ltd. (Beijing, China). All mice were kept in a specific-pathogen-free (SPF) animal house with free access to food and water, a 12 h light/dark cycle, and a constant temperature environment of 24°C. All animal experiments were approved by the Experimental Animal Ethics Committee of Tianjin University of Traditional Chinese Medicine, Tianjin, China (TCM-LAEC2018032). After 1 week of acclimatization to the home cage, the mice were randomly divided into five groups (*n* = 10): Normal Control group (Control), UV irradiated Model group (Model), blank matrix preparation group (Base), MQEF Low-dose (0.67 mg/g) group (L), MQEF (1.33 mg/g) high-dose group (H). The hairs on the back of the mice were removed by electric shaver. The back hair of the mice was shaved with an electric razor, and the gel corresponding to each group was applied to the exposed skin of the mice. UV radiometer (SS-03AB, Sigma) was used to irradiate the mice after the gel was completely absorbed. Mice were irradiated 5 times a week for 9 weeks and the control group did not do any treatment. The irradiation dose for the first week was the minimum erythema amount (MED): UVB: 0.07 J/cm^2^ and UVA: 0.7 J/cm^2^, followed by a steady increase in the weekly irradiation dose to a total radiation dose of 9.45 J/cm^2^ for UVB and 94.5 J/cm^2^ for UVA at a distance of 30 cm. After the experiment, the mice were anesthetized and the elasticity and wrinkles of the dorsal skin were examined. Subsequently, the mice were executed and the dorsal skin was removed for histological and western blot analysis.

### Determination of skin moisture content, sensitivity, pigmentation, wrinkle and elasticity

The skin moisture tester (Cornemeter CM 825, Courage and Khazaka, Germany) was used to measure the moisture content of the skin of mice. Specifically, a probe was placed against the back of the skin of the mice and the moisture content was analyzed by measuring the skin capacitance to evaluate the condition of the skin barrier. The mice were immobilization on a table and photographed using a Tissue Viability Imager (TiVi) 700 (WheelsBridge AB, Sweden). Skin sensitivity, pigmentation and wrinkle formation were analyzed using the TiVi 700 software package.

The skin lifting test was referred to as the skin elasticity test method of Tsukahara (Tsukahara et al., 2005). After anesthesia, the mouse skin was lifted from the midline as far as possible with the thumb and forefinger until the hind limbs just touched the table, which lasted for 1 s and then released, and the time required for the skin to recover to the original state was recorded immediately.

### Histopathological and immunofluorescence observation

The fixed skin tissue samples obtained from mice were embedded in paraffin blocks following euthanasia. Paraffin samples were dewaxed and washed in different concentrations of xylene and ethanol, the skin tissue structure of mice was investigated with Hematoxylin and Eosin (H&E) staining, collagen fiber of mouse skin tissue was observed with Masson staining, and elastic fiber of mouse skin tissue was inspected with Resorcinol-Fuchsin (Weigert) staining. During immunofluorescence staining, the sections were placed in a repair box filled with EDTA antigen repair buffer for antigen repair. The sections were placed in Nrf2 primary antibody and incubated overnight at 4°C. The corresponding secondary antibody was added after cleaning the next day and stained with 4′, 6-diamidine-2-phenylindole (DAPI) to ensure nuclear localization. The prepared sections were sealed and labeled with cover glass. Histopathological sections were scanned using 3D-HISTech digital pathology scanner (Pannoramic MIDI, Hungary) and analyzed using Case Viewer and ImageJ software.

### Statistical analysis

All experiments were repeated at least 3 times with representative results being shown. A one-way ANOVA was used to identify significant differences between the treatments (*p* = 0.05).

## Results

### UHPLC-Q-TOF-MS profile of MQEF

**FIGURE 1:**
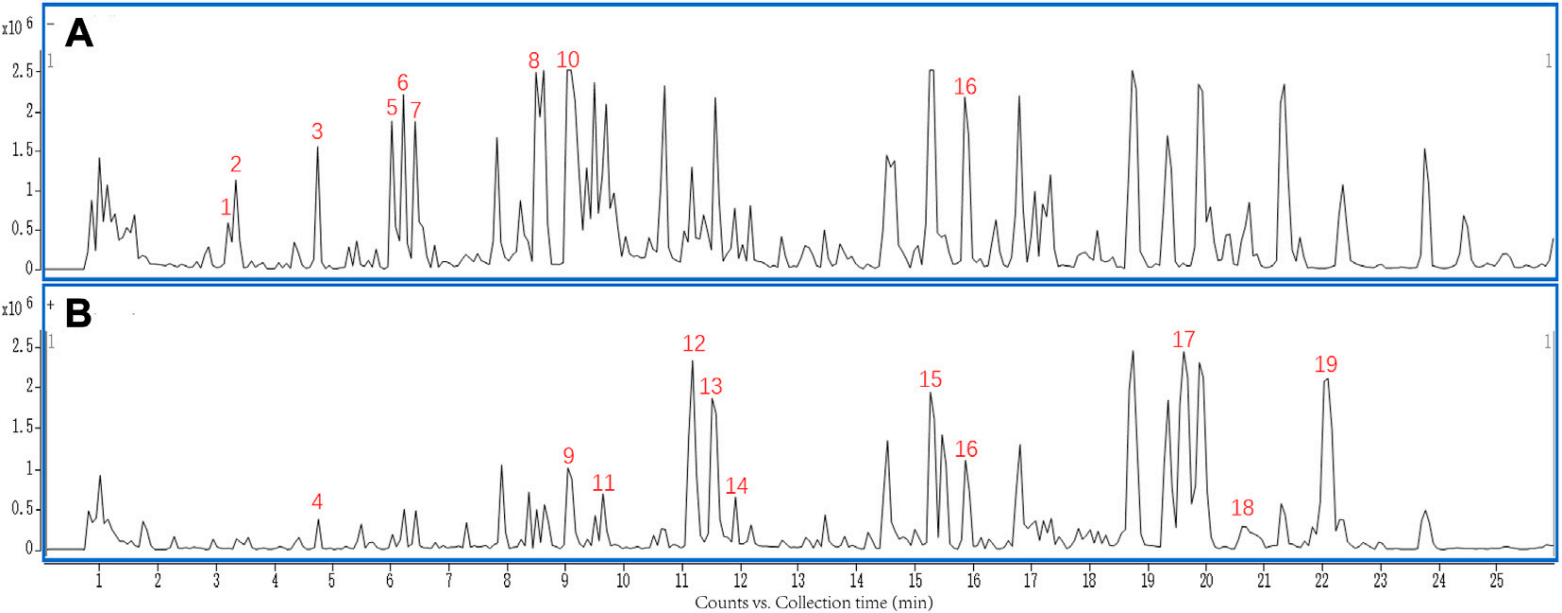
The chemical base peak intensity chromatogram (BPC) of compounds characterization of Modified Qing’e Formula (MQEF) extracts in positive ion mode and negative ion mode determined by UHPLC-Q-TOF-MS. **(A)** Negative ion modes. **(B)** Positive ion modes.

The MQEF extracts were analyzed by using UHPLC-Q-TOF-MS. As shown in Figure 1, the chemical base peak intensity chromatogram of MQEF extracts was based on the positive and negative ion modes of UHPLC-Q-TOF -MS. A total of 19 compounds were tentatively identified based on their retention times and mass fragmentation, the details were listed in Table 1.

**TABLE 1:** Chemical constituents identified of Modified Qing’e Formula (MQEF) extracts.

No.	Identification	Time	Elemental composition	Calculated mass	m/z	Mass error (ppm)	Origin
1	Salvianic acid A	3.19	C_9_H_10_O_5_	198.0528	[M-H]^-^197.0457	3.55	DS
2	Geniposidic acid	3.33	C_16_H_22_O_10_	374.1213	[M-H]^-^373.1124	4.34	DZ
3	Chlorogenic acid	4.74	C_16_H_18_O_9_	354.0951	[M-H]^-^353.0869	−1.13	DS
4	19-Hydroxycoumarin	4.74	C_9_H_6_O_3_	162.0317	[M + H]^+^163.0394	−8.77	
5	(+)-Piresil-4-O-β-D-glucopyraside	6.00	C_26_H_32_O_11_	520.1945	[M-H]^-^519.1870	0.36	DZ
					[M-H-Glc]^-^357.1338		
6	Psoralenoside/Isopsoralenoside	6.21	C_17_H_18_O_9_	366.0951	[M-H]^-^365.0919	−11.21	BGZ
					[M-H-Glc]^-^203.0349		
7	Psoralenoside/Isopsoralenoside	6.41	C_17_H_18_O_9_	366.0951	[M-H]^-^365.0859	5.22	BGZ
					[M-H-Glc]^-^203.0327		
8	Rosmarinic acid	8.48	C_18_H_16_O_8_	360.0845	[M-H]^-^359.0735	10.42	DS
					[M-H-DSS]^-^161.0210		
9	Protocatechualdehyde	9.03	C_7_H_6_O_3_	138.0317	[M + H]^+^139.0398	-5.97	DS
					[M-H]^-^717.1496		
10	Salvianolic acid B	9.09	C_36_H_30_O_16_	718.1534	[2M-H]^-^1,435.3034	-4.87	DS
					[M-H-DSS]^-^519.0946		
11	7-Methoxycoumarin	9.63	C_10_H_8_O_3_	176.0473	[M + H]^+^177.0555	-4.97	
12	Psoralen	11.17	C_11_H_6_O_3_	186.0317	[M + H]^+^187.0396	-3.37	BGZ
13	Angelicin	11.51	C_11_H_6_O_3_	186.0317	[M + H]^+^187.0398	-4.43	BGZ
14	Corylin	11.91	C_20_H_16_O_4_	320.1049	[M + H]^+^321.1119	0.73	BGZ
15	Daidzein	15.26	C_15_H_10_O_4_	254.0579	[M + H]^+^255.0622	11.70	BGZ
					[M + H-H_2_O]^+^237.0487		
16	Bavachin	15.92	C_20_H_20_O_4_	324.1362	[M-H]^-^323.1260	8.92	BGZ
					[M + H]^+^325.1435		
17	Cryptotanshinone	19.61	C_19_H_20_O_3_	296.1412	[M + H]^+^297.1460	8.48	DS
					[M + Na]^+^319.1285		
18	Kaempferide	20.68	C_16_H_12_O_6_	300.0634	[M + H]^+^301.0735	-9.42	DZ
19	Tanshinone IIA	22.02	C_19_H_18_O_3_	294.1256	[M + H]^+^295.1344	-5.18	DS
					[M + Na]^+^317.1170		

DS: *Salvia miltiorrhiza* Bunge.; DZ: *Eucommia ulmoides* Oliv.; BGZ: *Psoralea corylifolia* L.

### MQEF protects HaCaT cells from UV-induced oxidative damage

**FIGURE 2:**
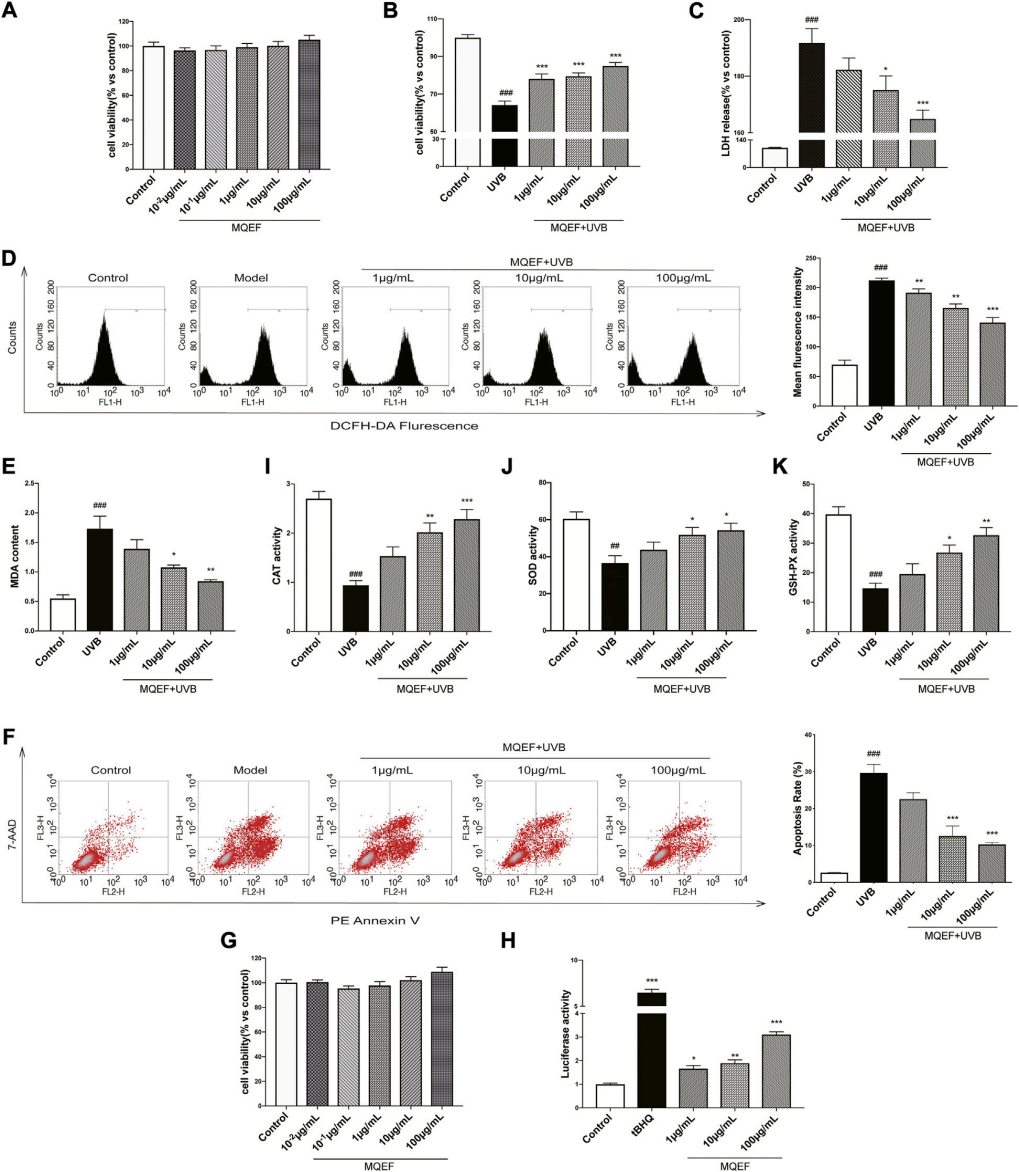
Modified Qing’e Formula (MQEF) protects HaCaT cells from UV-Induced oxidative damage. Cells were pretreated with MQEF at doses of 10^−2^, 10^−1^, 1, 10, and 100 μg/ml as well as no compound treatment for 24 h and then irradiated with UVB at a dose of 60 mJ/cm^2^. After irradiation, cells were continued to be cultured for 24 h and then processed for subsequent analysis. **(A)** Effect of MQEF treatment for 24 h on the cytotoxicity of HaCaT cells. **(B)** Effect of MQEF on HaCaT cell viability after UVB irradiation. **(C)** Effect of MQEF on lactate dehydrogenase (LDH) release of HaCaT cells after UVB irradiation to evaluate the degree of cell membrane damage. **(D)** Effects of MQEF on reactive oxygen species (ROS) content in HaCaT cells after UVB irradiation. **(E)** Effects of MQEF on malondialdehyde (MDA) content in HaCaT cells after UVB irradiation. **(F–H)** Effects of MQEF on catalase (CAT), superoxide dismutase (SOD) and glutathione peroxidase (GSH-Px) activity in HaCaT cells after UVB irradiation. **(I)** Effects of MQEF on apoptosis in HaCaT cells after UVB irradiation. **(J)** Effect of MQEF treatment for 24 h on cytotoxicity of HEK293T cells. **(K)** Effects of MQEF on ARE transcriptional activity of HEK293T cells. Data are mean ± SEM from 3 independent experiments. ^###^ *p* < 0.001 compared with the control group, ^##^ *p* < 0.01 compared with the control group; ^#^ *p* < 0.05 compared with the control group; ^***^ *p* < 0.001 compared with the model group; ^**^ *p* < 0.01 compared with the model group; ^*^ *p* < 0.05 compared with the model group.

We examined the cytotoxicity of MQEF on HaCaT cells with the CCK-8 kit. MQEF (concentration 10^−1^–100 μg/ml) did not show any significant cytotoxicity at 24 h (Figure 2A). Therefore, we fixed the concentration of MQEF at 1, 10, and 100 μg/ml for further cell-based experiments. As shown in Figures 2B,C, compared with the control group, cell viability was reduced by about 40% at 24 h after UVB irradiation, and LDH release was significantly increased, indicating that the cell photoaging model was successfully constructed. Compared with UVB group, the dose dependence of cell viability in MQEF group was significantly increased and the release amount of LDH was substantially decreased, indicating that MQEF could improve the cell viability of HaCaT after UVB radiation and improve the degree of cell membrane damage (Figures 2B,C).

Previous studies had demonstrated a link between increased intracellular ROS accumulation and the development of photoaging and skin cancer (Forrester et al., 2018). In order to understand whether MQEF has antioxidant ability to reduce UVB-mediated ROS production in HaCaT cells, ROS content in cells treated with different concentrations of MQEF was measured with DCFH-DA at the 24 h after UVB irradiation. UVB radiation alone triggered the accumulation of ROS in cells compared to control cells. This effect was significantly reduced in a concentration-dependent manner in MQEF pretreated cells (Figure 2D). Excessive ROS led to lipid peroxidation in the body and destroyed the structure of biofilm. It is obvious that the change of MDA content in HaCaT cells in each group was consistent with the ROS (Figure 2E). CAT, SOD and GSH-Px could decompose excessive ROS, reduce lipid peroxidation and improve the antioxidant capacity of the body. As expected, MQEF increased the activity of these antioxidant enzymes in UVB-irradiated cells (Figures 2F–H). Oxidative stress damage may also lead to apoptosis. The four quadrants in flow cytometry represented different cell states, and the apoptosis rate was determined by early and late apoptosis cells (Du et al., 2021). Apoptosis of HaCaT cells increased after UVB irradiation, but decreased significantly after MQEF (Figure 2I). These results indicated that MQEF had antioxidant capacity and protected HaCaT cells from light damage caused by UVB radiation.

### Potential mechanisms of MQEF protects HaCaT cells from oxidative damage

**FIGURE 3:**
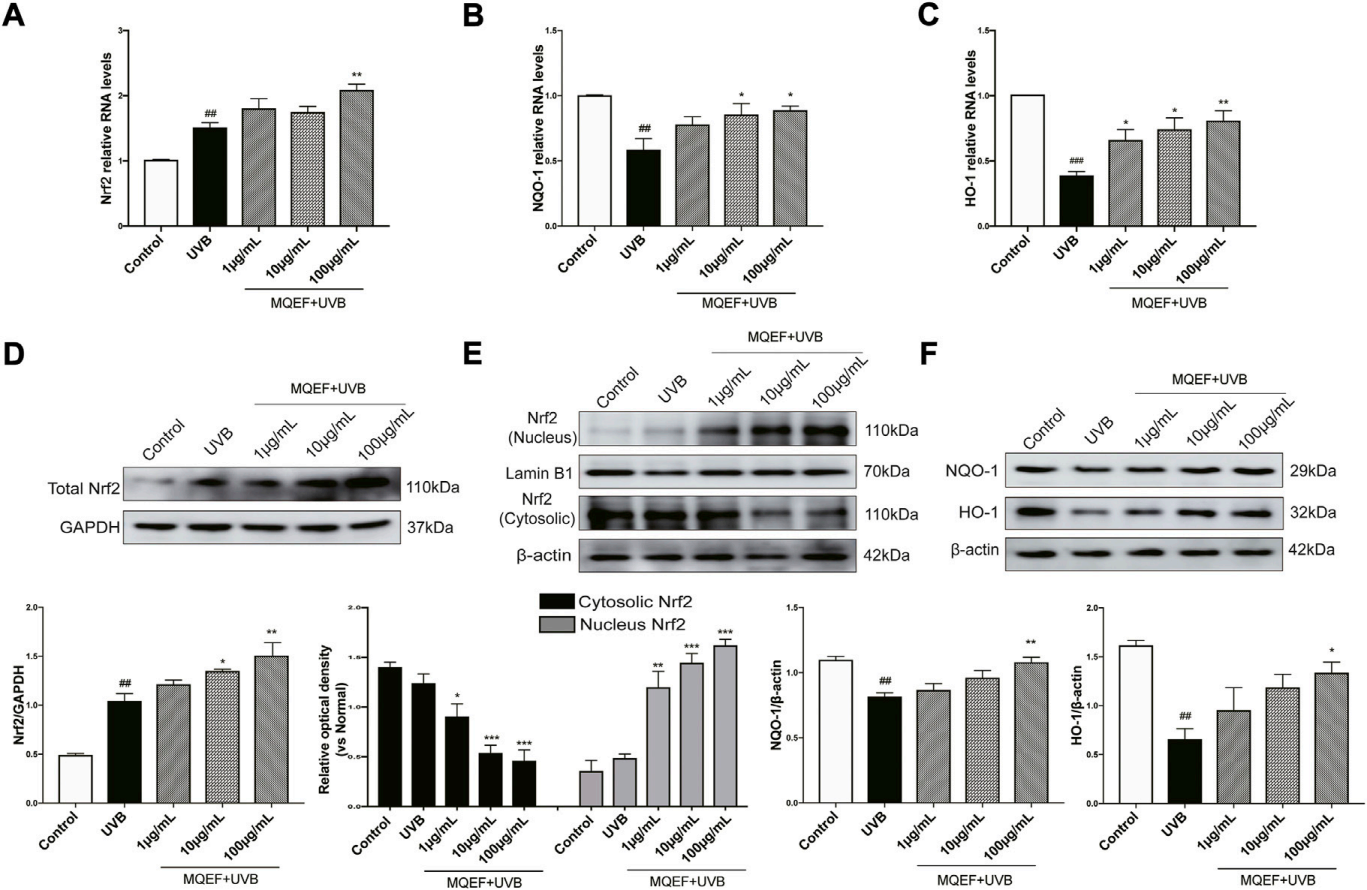
The potential mechanisms of Modified Qing’e Formula (MQEF) protect HaCaT cells from oxidative damage. Cells were pretreated with MQEF at doses of 1, 10, and 100 μg/ml as well as no compound treatment for 24 h and then irradiated with UVB at a dose of 60 mJ/cm^2^. After irradiation, cells were continued to be cultured for 24 h and then processed for subsequent analysis. **(A–C)** Effects of MQEF on mRNA expression of nuclear factor E2-related factor 2 (Nrf2), NAD(P)H quinone oxidoreductase 1 (NQO-1) and heme oxygenase-1 (HO-1) in HaCaT cells after UVB irradiation. **(D–F)** Effects of MQEF on protein expression of Nrf2, NQO-1 and HO-1 in HaCaT cells after UVB irradiation. Data are mean ± SEM from 3 independent experiments. ^###^ *p* < 0.001 compared with the control group, ^##^ *p* < 0.01 compared with the control group; ^#^ *p* < 0.05 compared with the control group; ^***^ *p* < 0.001 compared with the model group; ^**^ *p* < 0.01 compared with the model group; ^*^ *p* < 0.05 compared with the model group.

Nrf2/ARE activation was reported to be critical in regulating antioxidant gene expression (Ha and Boo, 2021). We then measured ARE activity levels after MQEF treatment and the results showed that MQEF significantly increased Nrf2/ARE luciflucase activity in a dose-dependent manner (Figures 2J,K). PCR results also showed transcriptional activation of Nrf2 in MQEF-treated cells at the 24th h after UVB irradiation (Figure 3A), which was consistent with western blotting results (Figure 3D). At the same time, MQEF significantly increased Nrf2 expression in the nucleus and decreased expression in the cytoplasm (Figure 3E). Nuclear translocation of Nrf2 mediated the induction of antioxidant genes such as HO-1 and NQO-1 to eliminate ROS and protect cells from oxidative damage. PCR (Figures 3B,C) and western blotting (Figure 3F) at 24 h after UVB irradiation showed that the mRNA and protein expressions of HO-1 and NQO-1 in HaCaT cells were significantly down-regulated. The expression of HO-1 and NQO-1 showed a trend of up-regulation after the administration of MQEF, and the high dose of MQEF had the best effect. We found that MQEF could activate Nrf2 to translocate from the cytoplasm to the nucleus and induce the expression of antioxidant genes, thus exerting antioxidant effects.

### MQEF prevents UV-induced oxidative damage which is Nrf2-dependent

**FIGURE 4:**
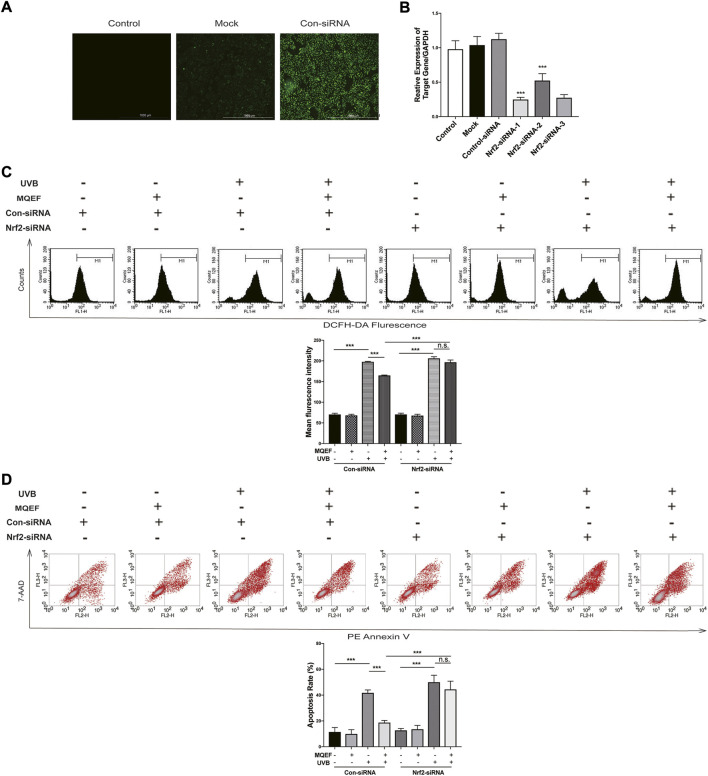
Modified Qing’e Formula (MQEF) prevents UV-induced oxidative damage which is nuclear factor E2-related factor 2 (Nrf2)-dependent. **(A)** After 4–7 h siRNA transfection, the effectiveness of siRNA transfection effect in HaCaT cells was observed by fluorescence. **(B)** After 4–7 h Nrf2-siRNA transfection, HaCaT cells were continued to be cultured for 48 h followed by reverse transcription-polymerase chain reaction (RT-PCR) to detect transfection efficiency. **(C)** Effect of MQEF on ROS content of HaCaT cells irradiated with UVB after 4–7 h of Nrf2-siRNA transfection. **(D)** Effect of MQEF on apoptosis of HaCaT cells irradiated with UVB after 4–7 h of Nrf2-siRNA transfection. Data are mean ± SEM from 3 independent experiments. ^###^ *p* < 0.001 compared with the control group, ^##^ *p* < 0.01 compared with the control group; ^#^ *p* < 0.05 compared with the control group; ^***^ *p* < 0.001 compared with the model group; ^**^ *p* < 0.01 compared with the model group; ^*^ *p* < 0.05 compared with the model group.

We used Nrf2-siRNA transfection to inhibit Nrf2 expression, thereby demonstrating that the protective effect of MQEF was mediated by Nrf2 activation. The siRNA reagent contains green fluorescent protein, and cells with green fluorescence under fluorescence microscope proved successful in transfection (Figure 4A). Figure 4B confirmed the successful knockdown of Nrf2 through PCR analysis, in which the transfection efficiency of Nrf2-siRNA-1 could reach more than 70%. The Nrf2-siRNA-1 sequence was selected for subsequent experiments (Figure 4B). Compared with the con-siRNA cells treated with UVB, pretreatment with MQEF effectively reduced the ROS production and apoptosis rate. However, for Nrf2-siRNA cells, MQEF failed to rescue the ascended ROS (Figure 4C) and apoptosis (Figure 4D). MQEF did not have such antioxidative effects on Nrf2-siRNA cells. These results suggested that Nrf2 was essential for the protective effects of MQEF under oxidative stress.

### MQEF protects mice from UV-induced skin damage

**FIGURE 5:**
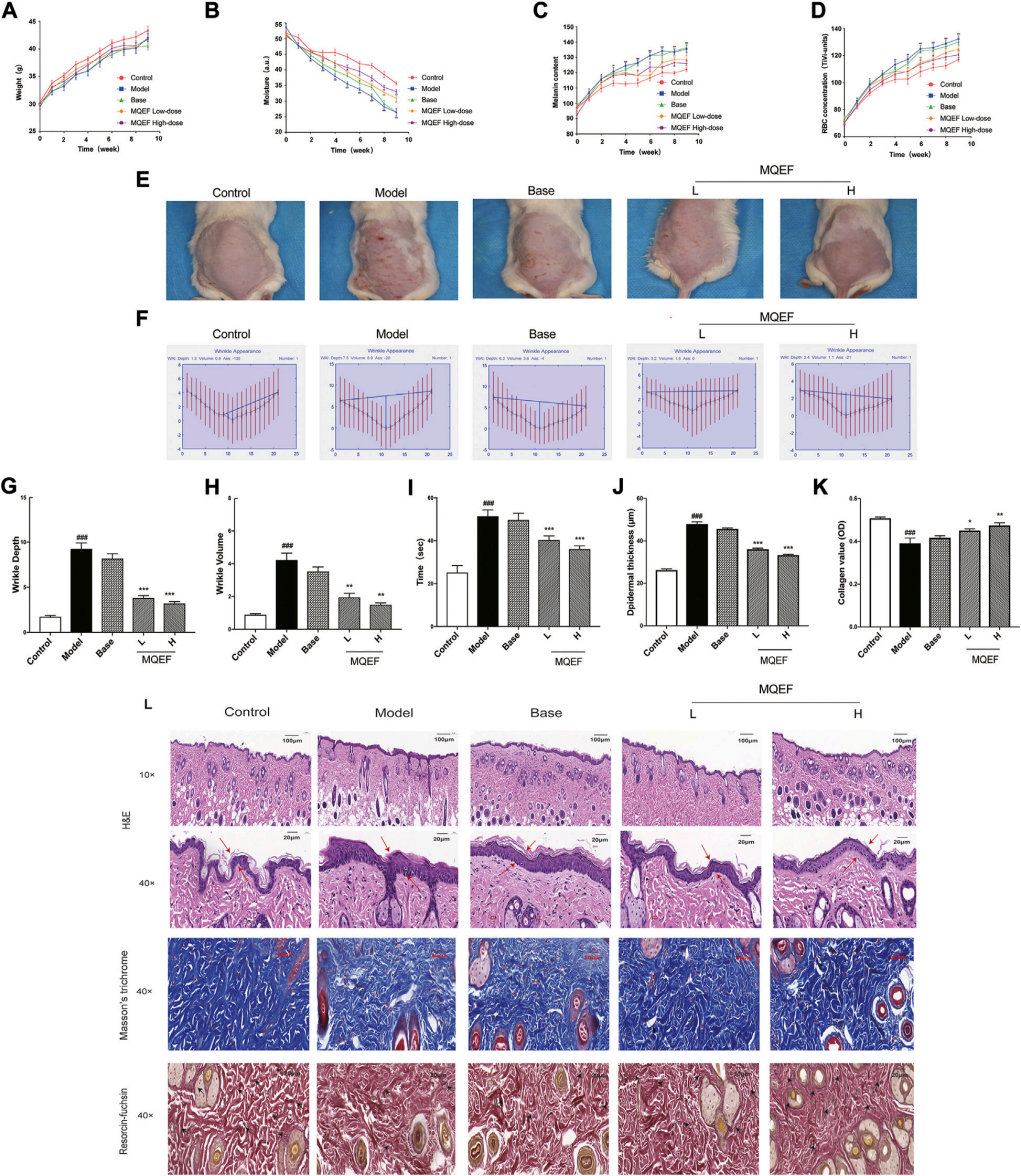
Modified Qing’e Formula (MQEF) protects mice from UV-Induced skin damage. Mice were treated topically with 0.67, 1.33 mg/g MQEF as well as no compound treatment, and the irradiation doses for the first week were minimal erythema (MED): UVB: 0.07 J/cm^2^ and UVA: 0.7 J/cm^2^, followed by a steady increase in the weekly irradiation dose to a total radiation dose of 9.45 J/cm^2^ for UVB and 94.5 J/cm^2^ for UVA. The irradiation was administered 5 times a week for 9 weeks **(A–D)** Representative broken lines of body weight, skin moisture, melanin content and sensitivity of mice within 9 weeks of UV irradiation. **(E)** Representative images of the skin morphological changes in each group after 9 weeks of UV irradiation. **(F–H)** The extent of wrinkle depth and volume was analyzed by image analysis system. **(I)** Changes of skin elasticity in each group after 9 weeks of UV radiation. **(J)** Quantification of the epidermal thickness in Hematoxylin and Eosin (H&E) staining. **(K)** Quantification of the collagen value in Masson’s staining. **(L)** H&E staining was used to evaluate the tissue structure of mouse skin. Observe the thickness of the *epidermis* by the double arrow; Masson’s staining was used to evaluate collagen fibers in mouse skin tissue. The elastic fiber was observed by arrows; Weigert staining was used to evaluate elastic fibers in mouse skin tissue. Data are mean ± SEM from 10 independent experiments. ^###^ *p* < 0.001 compared with the control group, ^##^ *p* < 0.01 compared with the control group; ^#^ *p* < 0.05 compared with the control group; ^***^ *p* < 0.001 compared with the model group; ^**^ *p* < 0.01 compared with the model group; ^*^ *p* < 0.05 compared with the model group.

In this study, ICR mice were exposed to UV irradiation, and MQEF was applied topically for 9 weeks. At the end of the experiment, no considerable differences were observed in the body weights of the mice in all five groups (Figure 5A). In the 9 weeks of modeling, it was obvious that the back skin of the control group mice was smooth and elastic; The skin of the model group showed photoaging characteristics such as skin thickening, rough relaxation, telangiectasia, lack of elasticity and thick and deep wrinkles; Compared with the model group, the roughness and dryness of the skin in the low dose group and the high dose group were improved to some extent, and the low dose group and the high dose group had a stronger effect on the improvement of the skin (Figure 5E). Skin hydration was important for maintaining skin health and protecting against various external stimuli (Doge et al., 2017). The present results showed that skin moisture in mice after UV exposure gradually decreased from week 3, and topical application of MQEF could alleviate water loss (Figure 5B). As shown in Figures 5C,D, the melanin content and sensitivity in the skin of mice in the model group became the highest from week 2, and after week 5, the melanin content in the MQEF group decreased significantly compared with that in the model group. These results suggest that MQEF could improve skin barrier protection in photoaging mice.

The process of wrinkle formation and skin elasticity may indicate the extent of skin damage (Hara et al., 2017). The levels of wrinkle formation and elasticity were evaluated in the present study. In Figures 5E,F, deep coarse wrinkles appeared in mice following UV exposure, which was attenuated following MQEF administration. The wrinkle depth (Figure 5G), wrinkle volume (Figure 5H) and skin elasticity (Figure 5I) were significantly increased in the UV-treated group compared to the control group. Notably, MQEF treatment markedly reduced these indicators, indicating its role in improving UV-induced skin damage. At the same time, compared with the model group, the changes of these indexes in the skin of the base group mice were not significant, suggesting that the application of blank gel matrix preparation did not affect the skin, and it was a completely the protective effect of MQEF on the photoaging mice.

The changes in histological properties in each group were examined. First, according to the results of H&E staining, the *epidermis* of the control group was thinner and the structure was intact. In the model group, the *epidermis* was significantly thickened, the junction between *epidermis* and dermis was flattened, the epidermal protrusions and dermal papilla were significantly reduced or even disappeared, and melanin was obviously increased and distributed unevenly. After MQEF was given, the situation improved (Figure 5L). Figure 5J quantified epidermal thickness, which was thinned by MQEF. Second, the plastic state of the collagen fibers was examined using Masson’s staining in each group (Figure 5L), statistical analysis showed that MQEF injection increased the density of the collagen fibers present in the model group (Figure 5K). Elastic fiber bundles could be observed by Weigert staining to evaluate skin elasticity. Compared with the control group, elastic fiber bundles in the dermis of mice in the model group were obviously broken and curly (Figure 5L). With the increase in dosage, the state of collagen fiber in MQEF group was significantly improved. Taken together, these results suggested that MQEF mitigated UV-induced skin histological damage in mice.

### MQEF inhibits UV-induced oxidative damage in mice skin

**FIGURE 6:**
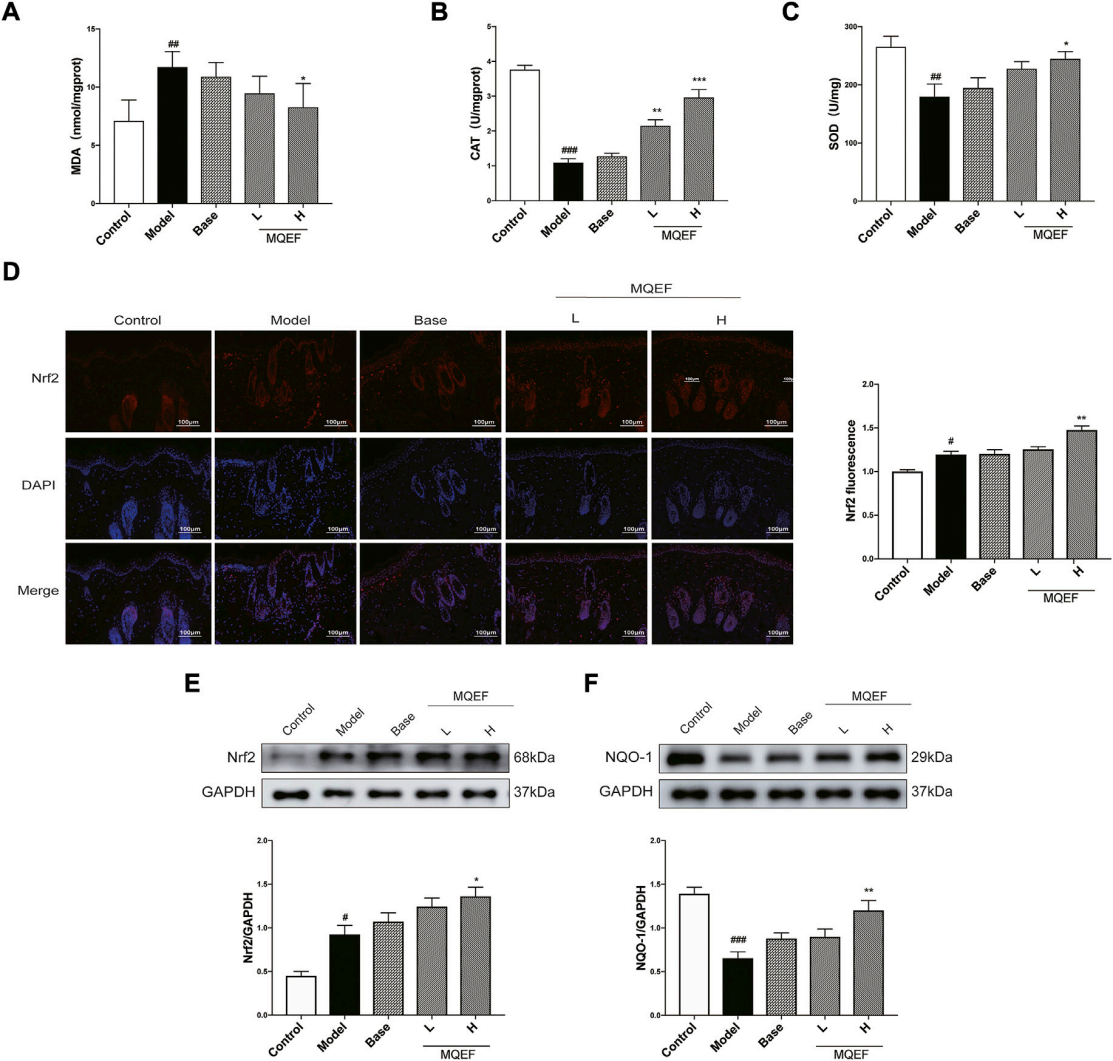
Modified Qing’e Formula (MQEF) inhibits UV-induced oxidative damage in mice skin. **(A)** The MDA content in serum of mice. **(B,C)** The catalase (CAT), superoxide dismutase (SOD) activity in the serum of mice. **(D)** Immunofluorescence staining was used to determine the subcellular localization of nuclear factor E2-related factor 2 (Nrf2), and the fluorescence intensity was analyzed and counted by ImageJ software. **(E,F)** Western blot analysis was performed to evaluate the protein expression levels of Nrf2 and NAD(P)H quinone oxidoreductase 1 (NQO-1). ImageJ software was used for analysis and statistical calculation. Data are mean ± SEM from 3 independent experiments. ^###^ *p* < 0.001 compared with the control group, ^##^ *p* < 0.01 compared with the control group; ^#^ *p* < 0.05 compared with the control group; ^***^ *p* < 0.001 compared with the model group; ^**^ *p* < 0.01 compared with the model group; ^*^ *p* < 0.05 compared with the model group.

Previous studies had proved that the oxidative stress induced by UVB irradiation was related to lipid peroxidation. MDA was the final and most important product of polyunsaturated fatty acid peroxidation, and the degree of oxidative stress could be assessed by evaluating MDA (Kong and Xu, 2020). Obviously, the content of MDA in the model group increased by > 1.5 times compared with the control group, and the dose-dependent reduction of MDA production by MQEF (Figure 6A). We then investigated the activity of antioxidant enzymes, including CAT and SOD, which are markers of oxidative stress. Reduced CAT and SOD levels were observed in the model group compared with the control group, while treatment with MQEF increased their activity (Figures 6B,C). These findings suggest that MQEF may increase antioxidant levels and reduce UV-induced lipid peroxidation, thereby protecting mice from UV-induced oxidative damage.

To further uncover the molecular mechanisms involved in the regulation of antioxidant enzymes, we evaluated the expression of Nrf2, a transcription factor that is a key factor in the activation of antioxidant systems. For 9 weeks of application administration and UV radiation, we observed that UV triggered the antioxidant defense system in the skin and stimulated Nrf2 expression, while MQEF treatment further enhanced Nrf2 expression level, indicating that MQEF was a good activator of Nrf2 (Figure 6E). After Nrf2 was activated, it entered the nucleus and affected the expression of downstream antioxidant enzymes. It was observed that the expression of NQO-1 decreased significantly under UV irradiation, while the level of NQO-1 recovered significantly after MQEF treatment (Figure 6F). Notably, the fluorescence intensity expression of Nrf2 was consistent with western blotting results. Immunofluorescence staining showed that the fluorescence intensity of Nrf2 in the nucleus increased after UV irradiation, while MQEF pretreatment significantly promoted the nuclear translocation of Nrf2 (Figure 6D). These data suggested that MQEF may play an important role in combating oxidative skin damage by promoting Nrf2 transcription into the nucleus to mediate the expression of antioxidant genes.

## Discussion

UV irradiation is the predominant cause of skin photodamage. Over time, photoaging may lead to actinic keratosis and skin cancer (Xian et al., 2017; Guo et al., 2020). According to the report, UV-mediated oxidative stress is the key factor in the pathogenesis of photoaging (Wang et al., 2019). Excessive reactive oxygen species are produced during skin photoaging. When the body cannot remove ROS in time, the redox system will be unbalanced, causing oxidative damage to intracellular nucleic acids, lipids and proteins, eventually triggering apoptosis and skin tissue damage (Fitsiou et al., 2021; Kim et al., 2022). Therefore, it is imperative to use safe and effective phytochemicals to protect the skin from these harmful effects. In this study, UVB-induced HaCaT cell damage was used to establish an *in vitro* model, and UV-irradiated mice dorsal skin was used as an *in vivo* model of skin photoaging. We found that MQEF acts as an antioxidant, greatly mitigating the harmful effects of UV induced *in vitro* and *in vivo* models.

Obviously, MQEF could improve HaCaT cell viability and cell membrane integrity after UVB irradiation. Meanwhile, local application of MQEF in photoaging mice significantly improved the pathological state of skin tissue, increased skin moisture content and elasticity, decreased skin sensitivity, and inhibited the occurrence of pigmentation and wrinkles. This directly indicated the potential value of MQEF in the prevention of skin injury. Previous research had shown that the three traditional Chinese medicines of MQEF had a variety of pharmacological effects, including antioxidant (He et al., 2014), antibacterial (Bai et al., 2015), anti-inflammatory (Ren et al., 2020) and anti-tumor (Chen et al., 2017; XD et al., 2019). Hence, we speculated that the photoprotective effect of MQEF was derived from its antioxidant properties. UVB exposure induces ROS production in HaCaT cells and initiates skin cell apoptosis, while MQEF provided protection against UVB-induced skin cell death by removing accumulated ROS and fighting oxidative stress. ROS acts on lipids to produce a peroxidation reaction, and the oxidation end product is MDA, which can cause the cross-linking polymerization of proteins, nucleic acids and other life macromolecules, and has cytotoxicity (Ahmed and Schenk, 2017; Gu et al., 2020). The body activates its antioxidant enzyme defense system upon stimulation to eliminate excess ROS and protect cells from oxidative stress and damage (Farhat et al., 2018; Perez et al., 2018; Ho et al., 2021). Thus, the activity levels of these enzymes can be used as markers of oxidative stress (Pudlarz et al., 2020). In this study, UV-exposed mice showed decreased SOD and CAT levels and increased MDA levels, which was consistent with previous studies, and MQEF could reverse this trend. Similarly, the expression changes of antioxidant enzymes and the MDA *in vitro* photoaging model were consistent with that *in vivo* model, and MQEF showed the same therapeutic effect.

The transcription factor Nrf2 is a key regulator of oxidative stress, and the Nrf2 pathway is an important antioxidant pathway involved in UV-induced skin damage (Rojo de la Vega et al., 2017; Ikehata and Yamamoto, 2018). Under normal physiological conditions, Kelch-like ECH-associated protein-1(Keap1) bound to Nrf2 and remained in the cytoplasm (Wang et al., 2020). However, under oxidative conditions, elevated ROS levels promote the dissociation of Nrf2 and Keap1 (Schafer and Werner, 2015; Panda et al., 2022). Dissociated Nrf2 is transferred to the nucleus and bound to the ARE, which subsequently regulates the expression of downstream antioxidant genes such as HO-1 and NQO1 to repair UV-induced skin damage (Xian et al., 2019; Alcaraz and Ferrandiz, 2020). Therefore, activation of Nrf2 is considered as a new and effective molecular strategy for skin photoprotection (Kim et al., 2011). Our results showed that MQEF treatment for 24 h increased Nrf2 mRNA and protein expression, promoted Nrf2 translocation into the nucleus, and increased ARE luciferase activity. Similarly, MQEF treatment for 9 weeks increased Nrf2 protein expression in mouse skin, and immunofluorescence showed that MQEF mediated the translocation of cell solute Nrf2 to the nucleus. NQO-1, a widely distributed FAD-dependent flavoprotein, could accelerate obligatory two-electron reductions of nitroaromatics, quinones, azo dyes, and quinonimines (Zhao et al., 2018; Li et al., 2019). NQO-1 exerts highly effective antioxidant functions and performs a cytoprotective role. HO-1 is a phase II detoxifying enzyme that converts heme to bilirubin, which acts as a powerful antioxidant that protects cells from oxidative damage and death (Rehman et al., 2020; Kerns et al., 2020). Besides, the sustained increase in oxidative stress mediated by ROS resulted in decreased cellular NQO-1 and HO-1 levels (Jeayeng et al., 2017; Kobaisi et al., 2019). This was consistent with previous studies that UV radiation reduced the expression of NQO-1 or HO-1 in mice skin or HaCaT cells, while MQEF stimulated the transcription of NQO-1 and HO-1 in a dose-dependent manner and increased their protein expression. Therefore, we concluded that MQEF could mediate nuclear localization and transcriptional activation of Nrf2, and induce the expression of NQO-1 and HO-1 to exert antioxidant effects. We further conducted Nrf2 knockdown studies to determine that Nrf2 activation is critical for MQEF mediated antioxidant protein expression. We showed that MQEF treatment could not reduce the occurrence of UVB-induced ROS and apoptosis in Nrf2-siRNA transfected cells, but MQEF treatment could significantly inhibit the accumulation of ROS and apoptosis induced by UVB in con-siRNA cells. This demonstrated that in the absence of Nrf2, MQEF could not exert antioxidant effects against UV-induced oxidative stress damage. In summary, our study found that MQEF protects against UV-induced skin oxidative damage *via* the activation of Nrf2/ARE defensive pathway. However, for *in vitro* experiments, we only selected a single UVB-induced oxidative damage model of HaCaT cells, and lacked studies on UVA and other skin cell models, which makes the study limited. More experimental models should be added to our future studies to enable a comprehensive study of the role of MQEF in anti-skin aging.

The various chemical components identified in MQEF extracts include coumarins, flavonoids, phenolic compounds, glycosides and terpenoids. The diverse compounds in MQEF contribute significantly to the biological functions of MQEF. Chlorogenic acid (CGA) has been demonstrated to have a variety of physiological properties, including anti-inflammatory, antioxidant, immunomodulatory, anti-bacterial and antitumor effects (Naveed et al., 2018), and it is widely recognized for its role in modulating skin function, possibly as a bioactive component of MQEF (Lee et al., 2021). CGA photoprotection protects HDF cells from UVA-induced photoaging by reducing the accumulation of UVA-induced ROS, mitigating DNA damage, inhibiting the degradation of collagen and enhancing the synthesis of collagen (Xue et al., 2022). Previous studies have shown that CGA reduces radiation-induced apoptosis and DNA damage by activating Nrf2 (Yin et al., 2022). Meanwhile, CGA can increase cellular antioxidant capacity by enhancing Nrf2 nuclear accumulation to protect HaCaT cells from airborne particulate matter (PM) (Ho et al., 2021). Corylin in MQEF may serve as a putative bioactive component of MQEF. Corylin is a flavonoid compound that is known to have antioxidant, anti-inflammatory and anti-proliferative effects (Zheng et al., 2019). Our previous studies have shown that Corylin has significant antioxidant stress damage effects, reducing UV-induced skin photodamage by activating Nrf2 expression (Li et al., 2022). In addition, Corylin treatment attenuated atherosclerotic lesions in apolipoprotein E (ApoE)-deficient mice by reducing ROS production and vascular cell adhesion protein-1 (VCAM-1) expression (Chen et al., 2020). Recent studies have found that Corylin alleviates senescence in HUVECs by inhibiting the mTOR pathway (Wang et al., 2022). These previous researches further supported our results that MQEF protects against UV-induced skin oxidative damage *via* the activation of Nrf2/ARE defensive pathway. Absolutely, due to the complexity of the bioactive components of MQEF, other components may also have important contributions, the qualitative and quantitative analysis of the bioactive components of MQEF remains challenging and requires further investigation.

## Conclusion

In conclusion, this study illustrated that MQEF could attenuate UV-induced oxidative stress damage in the skin, and its mechanism of action may be related to the activation and nuclear translocation of Nrf2. MQEF has promising applications in protecting skin cells from UV radiation-induced damage and premature skin aging.

## Data Availability

The original contributions presented in the study are included in the article/supplementary material, further inquiries can be directed to the corresponding authors.

## References

[B1] Ahmed F. Schenk P. M. (2017). UV-C radiation increases sterol production in the microalga Pavlova lutheri. Phytochemistry 139, 25–32. 10.1016/j.phytochem.2017.04.002 28407491

[B2] Alcaraz M. J. Ferrandiz M. L. (2020). Relevance of Nrf2 and heme oxygenase-1 in articular diseases. Free Radic. Biol. Med. 157, 83–93. 10.1016/j.freeradbiomed.2019.12.007 31830562

[B3] Bai M. M. Shi W. Tian J. M. Lei M. Kim J. H. Sun Y. N. (2015). Soluble epoxide hydrolase inhibitory and anti-inflammatory components from the leaves of Eucommia ulmoides Oliver (duzhong). J. Agric. Food Chem. 63, 2198–2205. 10.1021/acs.jafc.5b00055 25679330

[B4] Chen C. C. Li H. Y. Leu Y. L. Chen Y. J. Wang C. J. Wang S. H. (2020). Corylin inhibits vascular cell inflammation, proliferation and migration and reduces atherosclerosis in ApoE-deficient mice. Antioxidants (Basel) 9, E275. 10.3390/antiox9040275 32218307 PMC7222202

[B5] Chen C. H. Hwang T. L. Chen L. C. Chang T. H. Wei C. S. Chen J. J. (2017). Isoflavones and anti-inflammatory constituents from the fruits of Psoralea corylifolia. Phytochemistry 143, 186–193. 10.1016/j.phytochem.2017.08.004 28825980

[B6] Chen X. Yang C. Jiang G. (2021). Research progress on skin photoaging and oxidative stress. Postepy Dermatol. Alergol. 38, 931–936. 10.5114/ada.2021.112275 35125996 PMC8802961

[B7] Doge N. Avetisyan A. Hadam S. Pfannes E. K. B. Rancan F. Blume-Peytavi U. (2017). Assessment of skin barrier function and biochemical changes of *ex vivo* human skin in response to physical and chemical barrier disruption. Eur. J. Pharm. Biopharm. 116, 138–148. 10.1016/j.ejpb.2016.12.012 28012990

[B8] Du Z. Y. Shu Z. L. Li L. Song X. M. Ma X. L. Liao L. X. (2021). Baoyuan decoction alleviates myocardial infarction through the regulation of metabolic dysfunction and the mitochondria-dependent caspase-9/3 pathway. AHM 1, 49–58. 10.1097/HM9.0000000000000003

[B9] Farhat Z. Browne R. W. Bonner M. R. Tian L. Deng F. Swanson M. (2018). How do glutathione antioxidant enzymes and total antioxidant status respond to air pollution exposure? Environ. Int. 112, 287–293. 10.1016/j.envint.2017.12.033 29324239 PMC5899033

[B10] Fitsiou E. Pulido T. Campisi J. Alimirah F. Demaria M. (2021). Cellular senescence and the senescence-associated secretory phenotype as drivers of skin photoaging. J. Invest. Dermatol. 141, 1119–1126. 10.1016/j.jid.2020.09.031 33349436

[B11] Forrester S. J. Kikuchi D. S. Hernandes M. S. Xu Q. Griendling K. K. (2018). Reactive oxygen species in metabolic and inflammatory signaling. Circ. Res. 122, 877–902. 10.1161/CIRCRESAHA.117.311401 29700084 PMC5926825

[B12] Fotakis G. Timbrell J. A. (2006). *In vitro* cytotoxicity assays: Comparison of LDH, neutral red, MTT and protein assay in hepatoma cell lines following exposure to cadmium chloride. Toxicol. Lett. 160, 171–177. 10.1016/j.toxlet.2005.07.001 16111842

[B13] Gu Y. Han J. Jiang C. Zhang Y. (2020). Biomarkers, oxidative stress and autophagy in skin aging. Ageing Res. Rev. 59, 101036. 10.1016/j.arr.2020.101036 32105850

[B14] Guo Y. J. Pan W. W. Liu S. B. Shen Z. F. Xu Y. Hu L. L. (2020). ERK/MAPK signalling pathway and tumorigenesis. Exp. Ther. Med. 19, 1997–2007. 10.3892/etm.2020.8454 32104259 PMC7027163

[B15] Ha J. W. Boo Y. C. (2021). Siegesbeckiae herba extract and chlorogenic acid ameliorate the death of HaCaT keratinocytes exposed to airborne particulate matter by mitigating oxidative stress. Antioxidants (Basel) 10, 1762. 10.3390/antiox10111762 34829633 PMC8615115

[B16] Hara Y. Hirao T. Iwai I. (2017). Facial expression under stiff stratum corneum leads to strain concentrations, followed by residual wrinkle formation. Int. J. Cosmet. Sci. 39, 66–71. 10.1111/ics.12349 27309128

[B17] Harris-Tryon T. A. Grice E. A. (2022). Microbiota and maintenance of skin barrier function. Science 376, 940–945. 10.1126/science.abo0693 35617415

[B18] He X. Wang J. Li M. Hao D. Yang Y. Zhang C. (2014). Eucommia ulmoides Oliv.: Ethnopharmacology, phytochemistry and pharmacology of an important traditional Chinese medicine. J. Ethnopharmacol. 151, 78–92. 10.1016/j.jep.2013.11.023 24296089

[B19] Ho C. C. Ng S. C. Chuang H. L. Wen S. Y. Kuo C. H. Mahalakshmi B. (2021). Extracts of Jasminum sambac flowers fermented by Lactobacillus rhamnosus inhibit H2O2 - and UVB-induced aging in human dermal fibroblasts. Environ. Toxicol. 36, 607–619. 10.1002/tox.23065 33270331

[B20] Hseu Y. C. Chang C. T. Gowrisankar Y. V. Chen X. Z. Lin H. C. Yen H. R. (2019). Zerumbone exhibits antiphotoaging and dermatoprotective properties in ultraviolet A-irradiated human skin fibroblast cells via the activation of Nrf2/ARE defensive pathway. Oxid. Med. Cell. Longev. 2019, 4098674. 10.1155/2019/4098674 31814875 PMC6878809

[B21] Ikehata H. Yamamoto M. (2018). Roles of the KEAP1-NRF2 system in mammalian skin exposed to UV radiation. Toxicol. Appl. Pharmacol. 360, 69–77. 10.1016/j.taap.2018.09.038 30268578

[B22] Jeayeng S. Wongkajornsilp A. Slominski A. T. Jirawatnotai S. Sampattavanich S. Panich U. (2017). Nrf2 in keratinocytes modulates UVB-induced DNA damage and apoptosis in melanocytes through MAPK signaling. Free Radic. Biol. Med. 108, 918–928. 10.1016/j.freeradbiomed.2017.05.009 28495448 PMC5546090

[B66] Kerns M. L. Miller R. J. Mazhar M. Byrd A. S. Archer N. K. Pinkser B. L. (2020). Pathogenic and therapeutic role for NRF2 signaling in ultraviolet light-induced skin pigmentation. JCI Insight. 5. 10.1172/jci.insight.139342 PMC7605539 33001866

[B23] Kim H. J. Zheng M. Kim S. K. Cho J. J. Shin C. H. Joe Y. (2011). CO/HO-1 induces NQO-1 expression via Nrf2 activation. Immune Netw. 11, 376–382. 10.4110/in.2011.11.6.376 22346778 PMC3275707

[B24] Kim M. Ha L. K. Oh S. Fang M. Zheng S. Bellere A. D. (2022). Antiphotoaging effects of damiana (turnera diffusa) leaves extract via regulation AP-1 and Nrf2/ARE signaling pathways. Plants (Basel) 11, 1486. 10.3390/plants11111486 35684259 PMC9182839

[B25] Kobaisi F. Fayyad N. Rezvani H. R. Fayyad-Kazan M. Sulpice E. Badran B. (2019). Signaling pathways, chemical and biological modulators of nucleotide excision repair: The faithful shield against UV genotoxicity. Oxid. Med. Cell. Longev. 2019, 4654206. 10.1155/2019/4654206 31485292 PMC6702832

[B26] Kong Y. H. Xu S. P. (2020). Juglanin administration protects skin against UVBinduced injury by reducing Nrf2dependent ROS generation. Int. J. Mol. Med. 46, 67–82. 10.3892/ijmm.2020.4589 32377697 PMC7255487

[B27] Krutmann J. Schikowski T. Morita A. Berneburg M. (2021). Environmentally-induced (extrinsic) skin aging: Exposomal factors and underlying mechanisms. J. Invest. Dermatol. 141, 1096–1103. 10.1016/j.jid.2020.12.011 33541724

[B28] Lee K. H. Do H. K. Kim D. Y. Kim W. (2021). Impact of chlorogenic acid on modulation of significant genes in dermal fibroblasts and epidermal keratinocytes. Biochem. Biophys. Res. Commun. 583, 22–28. 10.1016/j.bbrc.2021.10.057 34715497

[B29] Li J. Malakhova M. Mottamal M. Reddy K. Kurinov I. Carper A. (2012). Norathyriol suppresses skin cancers induced by solar ultraviolet radiation by targeting ERK kinases. Cancer Res. 72, 260–270. 10.1158/0008-5472.CAN-11-2596 22084399 PMC3251698

[B30] Li N. Liu T. Zhu S. Yang Y. Wang Z. Zhao Z. (2022). Corylin from Psoralea fructus (Psoralea corylifolia L.) protects against UV-induced skin aging by activating Nrf2 defense mechanisms. Phytother. Res. 36, 3276–3294. 10.1002/ptr.7501 35821646

[B31] Li X. Zhan J. Hou Y. Hou Y. Chen S. Luo D. (2019). Coenzyme Q10 regulation of apoptosis and oxidative stress in H2O2 induced BMSC death by modulating the nrf-2/NQO-1 signaling pathway and its application in a model of spinal cord injury. Oxid. Med. Cell. Longev. 2019, 6493081. 10.1155/2019/6493081 31915512 PMC6930770

[B32] Liu S. Pi J. Zhang Q. (2022). Signal amplification in the KEAP1-NRF2-ARE antioxidant response pathway. Redox Biol. 54, 102389. 10.1016/j.redox.2022.102389 35792437 PMC9287733

[B33] Ma H. Bell K. N. Loker R. N. (2021). qPCR and qRT-PCR analysis: Regulatory points to consider when conducting biodistribution and vector shedding studies. Mol. Ther. Methods Clin. Dev. 20, 152–168. 10.1016/j.omtm.2020.11.007 33473355 PMC7786041

[B34] Melo C. P. B. Saito P. Vale D. L. Rodrigues C. C. A. Pinto I. C. Martinez R. M. (2021). Protective effect of oral treatment with Cordia verbenacea extract against UVB irradiation deleterious effects in the skin of hairless mouse. J. Photochem. Photobiol. B 216, 112151. 10.1016/j.jphotobiol.2021.112151 33581679

[B35] Naveed M. Hejazi V. Abbas M. Kamboh A. A. Khan G. J. Shumzaid M. (2018). Chlorogenic acid (CGA): A pharmacological review and call for further research. Biomed. Pharmacother. 97, 67–74. 10.1016/j.biopha.2017.10.064 29080460

[B36] Panda H. Wen H. Suzuki M. Yamamoto M. (2022). Multifaceted roles of the KEAP1-NRF2 system in cancer and inflammatory disease milieu. Antioxidants (Basel) 11, 538. 10.3390/antiox11030538 35326187 PMC8944524

[B37] Perez S. Rius-Perez S. Tormos A. M. Finamor I. Nebreda A. R. Talens-Visconti R. (2018). Age-dependent regulation of antioxidant genes by p38α MAPK in the liver. Redox Biol. 16, 276–284. 10.1016/j.redox.2018.02.017 29567616 PMC5952885

[B38] Pudlarz A. M. Czechowska E. Karbownik M. S. Ranoszek-Soliwoda K. Tomaszewska E. Celichowski G. (2020). The effect of immobilized antioxidant enzymes on the oxidative stress in UV-irradiated rat skin. Nanomedicine 15, 23–39. 10.2217/nnm-2019-0166 31868116

[B39] Rehman M. U. Rashid S. Arafah A. Qamar W. Alsaffar R. M. Ahmad A. (2020). Piperine regulates nrf-2/keap-1 signalling and exhibits anticancer effect in experimental colon carcinogenesis in wistar rats. Biol. (Basel) 9, E302. 10.3390/biology9090302 PMC7565681 32967203

[B40] Ren Y. Song X. Tan L. Guo C. Wang M. Liu H. (2020). A review of the pharmacological properties of psoralen. Front. Pharmacol. 11, 571535. 10.3389/fphar.2020.571535 33013413 PMC7500444

[B65] Rojo de la Vega M. Krajisnik A. Zhang D. D. Wondrak G. T. (2017). Targeting NRF2 for improved skin barrier function and photoprotection: Focus on the achiote-derived apocarotenoid bixin. Nutrients. 9. 10.3390/nu9121371 PMC5748821 29258247

[B41] Schafer M. Werner S. (2015). Nrf2-A regulator of keratinocyte redox signaling. Free Radic. Biol. Med. 88, 243–252. 10.1016/j.freeradbiomed.2015.04.018 25912479

[B42] Shi X. Q. Chen G. Tan J. Q. Li Z. Chen S. M. He J. H. (2022). Total alkaloid fraction of Leonurus japonicus Houtt. Promotes angiogenesis and wound healing through SRC/MEK/ERK signaling pathway. J. Ethnopharmacol. 295, 115396. 10.1016/j.jep.2022.115396 35598796

[B43] Tian L. M. Peng Y. Ke D. Li H. Chen L. Zhang C. (2020). The effect of Yang Yan Qing E Wan on senescent phenotypes and the expression of beta-catenin and p16(INK4a) in human skin fibroblasts. J. Tissue Viability 29, 354–358. 10.1016/j.jtv.2020.06.001 32768331

[B44] Torrente L. DeNicola G. M. (2022). Targeting NRF2 and its downstream processes: Opportunities and challenges. Annu. Rev. Pharmacol. Toxicol. 62, 279–300. 10.1146/annurev-pharmtox-052220-104025 34499527

[B45] Tsukahara K. Moriwaki S. Hotta M. Fujimura T. Sugiyama-Nakagiri Y. Sugawara S. (2005). The effect of sunscreen on skin elastase activity induced by ultraviolet-A irradiation. Biol. Pharm. Bull. 28, 2302–2307. 10.1248/bpb.28.2302 16327169

[B46] Wakamori S. Taguchi K. Nakayama Y. Ohkoshi A. Sporn M. B. Ogawa T. (2022). Nrf2 protects against radiation-induced oral mucositis via antioxidation and keratin layer thickening. Free Radic. Biol. Med. 188, 206–220. 10.1016/j.freeradbiomed.2022.06.239 35753588

[B47] Wang M. Charareh P. Lei X. Zhong J. L. (2019). Autophagy: Multiple mechanisms to protect skin from ultraviolet radiation-driven photoaging. Oxid. Med. Cell. Longev. 2019, 8135985. 10.1155/2019/8135985 31915514 PMC6930764

[B48] Wang T. H. Tseng W. C. Leu Y. L. Chen C. Y. Lee W. C. Chi Y. C. (2022). The flavonoid corylin exhibits lifespan extension properties in mouse. Nat. Commun. 13, 1238. 10.1038/s41467-022-28908-2 35264584 PMC8907184

[B49] Wang T. Jian Z. Baskys A. Yang J. Li J. Guo H. (2020). MSC-derived exosomes protect against oxidative stress-induced skin injury via adaptive regulation of the NRF2 defense system. Biomaterials 257, 120264. 10.1016/j.biomaterials.2020.120264 32791387

[B50] Wu P. Y. Lyu J. L. Liu Y. J. Chien T. Y. Hsu H. C. Wen K. C. (2017). Fisetin regulates Nrf2 expression and the inflammation-related signaling pathway to prevent UVB-induced skin damage in hairless mice. Int. J. Mol. Sci. 18, E2118. 10.3390/ijms18102118 28994699 PMC5666800

[B51] Xd M. E. Cao Y. F. Che Y. Y. Li J. Shang Z. P. Zhao W. J. (2019). Danshen: A phytochemical and pharmacological overview. Chin. J. Nat. Med. 17, 59–80. 10.1016/S1875-5364(19)30010-X 30704625

[B52] Xian D. Gao X. Xiong X. Xu J. Yang L. Pan L. (2017). Photoprotection against UV-induced damage by skin-derived precursors in hairless mice. J. Photochem. Photobiol. B 175, 73–82. 10.1016/j.jphotobiol.2017.08.027 28865317

[B53] Xian D. Xiong X. Xu J. Xian L. Lei Q. Song J. (2019). Nrf2 overexpression for the protective effect of skin-derived precursors against UV-induced damage: Evidence from a three-dimensional skin model. Oxid. Med. Cell. Longev. 2019, 7021428. 10.1155/2019/7021428 31737172 PMC6815583

[B54] Xiong J. L. Cai X. Y. Zhang Z. J. Li Q. Zhou Q. Wang Z. T. (2022). Elucidating the estrogen-like effects and biocompatibility of the herbal components in the Qing' E formula. J. Ethnopharmacol. 283, 114735. 10.1016/j.jep.2021.114735 34637969

[B55] Xue N. Liu Y. Jin J. Ji M. Chen X. (2022). Chlorogenic acid prevents UVA-induced skin photoaging through regulating collagen metabolism and apoptosis in human dermal fibroblasts. Int. J. Mol. Sci. 23, 6941. 10.3390/ijms23136941 35805942 PMC9266774

[B56] Yang Y. P. Shuai B. Shen L. Xu X. J. Ma C. Lv L. (2015). Effect of Qing'e formula on circulating sclerostin levels in patients with postmenopausal osteoporosis. J. Huazhong Univ. Sci. Technol. Med. Sci. 35, 525–530. 10.1007/s11596-015-1464-8 26223921

[B57] Yin X. He X. Wu L. Yan D. Yan S. (2022). Chlorogenic acid, the main antioxidant in coffee, reduces radiation-induced apoptosis and DNA damage via NF-E2-Related factor 2 (Nrf2) activation in hepatocellular carcinoma. Oxid. Med. Cell. Longev. 2022, 4566949. 10.1155/2022/4566949 35958020 PMC9363170

[B58] Zembruski N. C. Stache V. Haefeli W. E. Weiss J. (2012). 7-Aminoactinomycin D for apoptosis staining in flow cytometry. Anal. Biochem. 429, 79–81. 10.1016/j.ab.2012.07.005Shi 22796502

[B59] Zhang J. M. Li J. Liu E. W. Wang H. Fan G. W. Wang Y. F. (2016). Danshen enhanced the estrogenic effects of Qing E formula in ovariectomized rats. BMC Complement. Altern. Med. 16, 181. 10.1186/s12906-016-1146-5 27339619 PMC4918020

[B60] Zhao P. Alam M. B. Lee S. H. (2018). Protection of UVB-induced photoaging by fuzhuan-brick tea aqueous extract via MAPKs/nrf2-mediated down-regulation of MMP-1. Nutrients 11, E60. 10.3390/nu11010060 30597920 PMC6357030

[B61] Zhao Z. Liu T. Zhu S. Yang Y. Wang Z. Ma H. (2021). Development and evaluation studies of Corylin loaded nanostructured lipid carriers gel for topical treatment of UV-induced skin aging. Exp. Gerontol. 153, 111499. 10.1016/j.exger.2021.111499 34329721

[B62] Zheng Z. G. Zhang X. Liu X. X. Jin X. X. Dai L. Cheng H. M. (2019). Inhibition of HSP90β improves lipid disorders by promoting mature SREBPs degradation via the ubiquitin-proteasome system. Theranostics 9, 5769–5783. 10.7150/thno.36505 31534518 PMC6735373

[B63] Zhong L. Huang F. Shi H. Wu H. Zhang B. Wu X. (2016). Qing'E formula alleviates the aging process in D-galactose-induced aging mice. Biomed. Rep. 5, 101–106. 10.3892/br.2016.667 27347412 PMC4906976

[B64] Zhu S. Zhao Z. Qin W. Liu T. Yang Y. Wang Z. (2022). The Nanostructured lipid carrier gel of Oroxylin A reduced UV-induced skin oxidative stress damage. Colloids Surf. B Biointerfaces 216, 112578. 10.1016/j.colsurfb.2022.112578 35636325

